# Well-Differentiated Papillary Mesothelial Tumor of the Scrotum with Suspicious Invasion

**DOI:** 10.3390/diagnostics14020169

**Published:** 2024-01-11

**Authors:** Soyoung Im, Je Mo Yoo, Uiju Cho

**Affiliations:** 1 Department of Pathology, St. Vincent’s Hospital, College of Medicine, The Catholic University of Korea, Seoul 06591, Republic of Korea; st9747@catholic.ac.kr; 2 Department of Urology, St. Vincent’s Hospital, College of Medicine, The Catholic University of Korea, Seoul 06591, Republic of Korea; albatrosyoo@naver.com

**Keywords:** mesothelioma, testicular hydrocele

## Abstract

Well-differentiated papillary mesothelial tumor (WDPMT) is a distinct form of mesothelioma with low malignant potential and is mostly found in the peritoneal cavity. It consists of mesothelial cells with papillary structure and bland cytology. We report a rare case of WDPMT with suspicious invasive foci in the tunica vaginalis. WDPMT with invasive foci is known to have a tendency for recurrence. Therefore, careful attention should be given to properly diagnosing and treating this rare entity.

**Figure 1:**
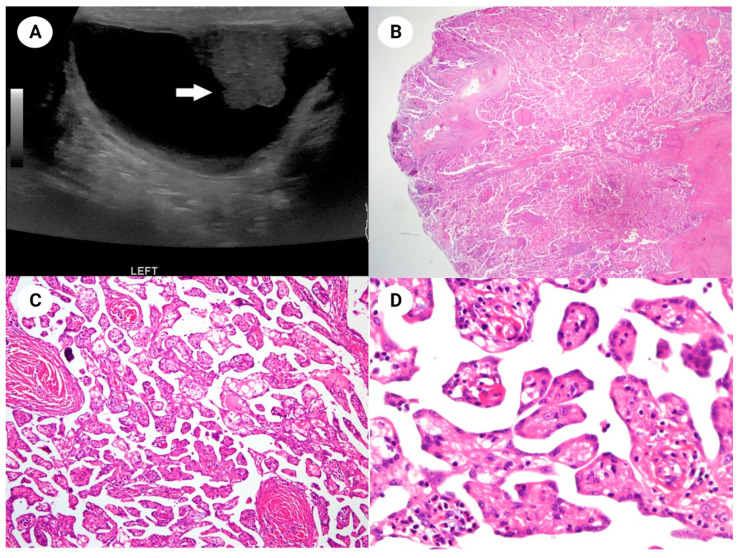
(**A**) A 22-year-old man presented with swelling in the left scrotum. Despite multiple attempts at fluid aspiration, the scrotal swelling recurred. Sonography revealed a protruding mass (arrow) measuring 2.2 × 1.5 cm within the hydrocele sac. Based on the clinical impression of hydrocele, a hydrocelectomy was performed. (**B**) Microscopically, the protruding mass in the tunica vaginalis consisted of well-developed papillae and thick fibrous stalks (H&E stain, ×12.5). (**C**) The entire mass exhibited branching papillary structures or blunt processes. Some stromal cores displayed loose fibrous or fibromyxoid stroma (H&E stain, ×100). (**D**) The papillary cores were lined with a single layer of uniform cuboidal mesothelial cells, characterized by central, rounded nuclei and a very low mitotic rate (H&E stain, ×400).

**Figure 2:**
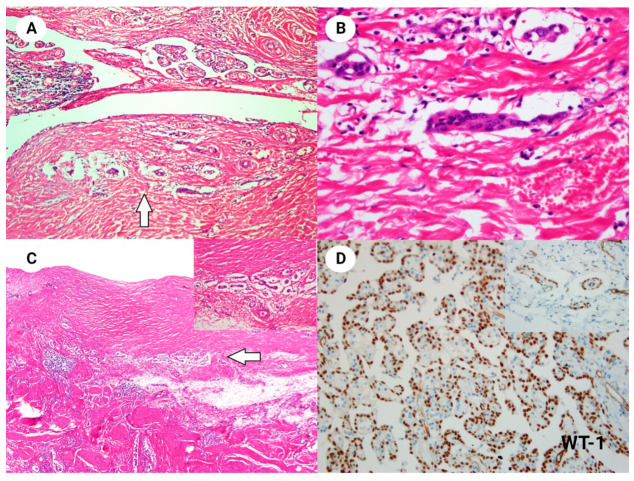
(**A**) Clusters of small glands were identified within the fibrous wall of the sac (arrow). These suspicious invasive foci were multiple and distributed along the sac wall (H&E stain, ×100). (**B**) The stromal glands exhibited mild cytologic atypia without mitotic activity. No stromal reactions, such as inflammation and desmoplasia, were observed around these glands (HE, ×400). (**C**) Multiple sites of suspicious invasive foci were identified in the specimen (arrow), all displaying bland cytologic features (H&E stain, ×100; inset ×400). (**D**) Immunohistochemical analysis revealed positive staining for the mesothelial cell marker WT-1 in both the main papillary mass and the small stromal glands (inset). Additionally, the tumor cells were positive for calretinin, cytokeratin 7, podoplanin and p16, while being negative for CEA, cytokeratin 20, EMA and MOC-31 (WT-1, magnification ×200; inset ×400). We conducted next-generation sequencing (NGS) to investigate the presence of a *BAP1* mutation, homozygous deletion of *CDKN2A*, or *BRAF* p.V600E mutation. However, our case did not exhibit alterations in these genes. No significant pathogenic mutations were identified in the NGS. Unfortunately, our NGS panel, consisting of more than 500 genes, did not include *TRAF7* and *CDC42*, reportedly in well-differentiated papillary mesothelial tumors of the peritoneum [1]. Based on these findings, a diagnosis of well-differentiated papillary mesothelial tumor with suspicious invasive foci was established. The patient received no adjuvant therapy and remains well without recurrence after 48 months of follow-up. Well-differentiated papillary mesothelioma (WDPMT) represents a distinct variant of mesothelial tumor characterized by low or no malignant potential, typically discovered incidentally within the peritoneal cavity. Although rare, it can manifest in paratesticular locations, such as the tunica vaginalis [2]. In our case, as others reported, WDPMT displayed mesothelial cells arranged in a papillary structure with bland cytology. The literature predominantly reports WDPMT as individual cases or in small case series [2,3,4,5,6,7,8]. Notably, there have been isolated case reports detailing WDPMT with invasive foci, with the first case series documenting 20 instances of WDPMT containing invasive foci [9]. This study suggested that WDPMTs with invasive foci may exhibit a propensity for multifocality and recurrence [9]. However, in a subsequent case report, a patient diagnosed with WDPMT, presenting invasive foci in the pleura, exhibited no recurrence over a follow-up period exceeding three years [10]. Another case series documented that, among 24 cases of WDPMT affecting the pleura, 10 exhibited submesothelial layer invasion. Unfortunately, the clinical course of this subgroup of patients was not thoroughly documented [11]. The 5th edition of the WHO classification of urinary and male genital tumors distinguishes well-differentiated papillary mesothelial tumor from mesothelioma, classifying it as benign. A key diagnostic criterion is the absence or minimal presence of stromal invasion [2,7]. Due to the limited number of reported cases, understanding the clinical course of WDPMT with suspicious invasion remains challenging. It is important to differentiate between a true invasion and a reactive mesothelial proliferation, which may not be part of WDPMT. However, this distinction is particularly challenging given the typically bland nature of mesothelial cells in WDPMT. Our case contributes evidence that focal or minimal invasion in WDPMT can still merit a diagnosis of WDPMT and may exhibit an indolent behavior. In the realm of male testicular and paratesticular tumors, their diversity is noteworthy, underscoring the necessity for meticulous preoperative examinations to establish precise diagnoses. Among the recently developed and advanced diagnostic modalities is contrast-enhanced ultrasonography (CEUS). CEUS offers both qualitative and quantitative parameters, presenting a promising approach for the differential diagnosis of testicular masses [12,13]. Furthermore, postoperative vigilance is imperative for accurately diagnosing and appropriately managing this rare pathology.

## Data Availability

Data are contained within the article.

## References

[1] Stevers M., Rabban J.T., Garg K., Van Ziffle J., Onodera C., Grenert J.P., Yeh I., Bastian B.C., Zaloudek C., Solomon D.A. (2019). Well-differentiated papillary mesothelioma of the peritoneum is genetically defined by mutually exclusive mutations in TRAF7 and CDC42. Mod. Pathol..

[2] Idrees M.T., Comperat E.M., Galateau-Salle F. (2022). Well-differentiated papillary mesothelial tumour. WHO Classification of Tumours: Urinary and Male Genital Tumours.

[3] Cabay R.J., Siddiqui N.H., Alam S. (2006). Paratesticular papillary mesothelioma: A case with borderline features. Arch. Pathol. Lab Med..

[4] Gan A.M.L., Plantinga P., Punjani N., Hussey A., Power N. (2018). Images—Well-differentiated papillary mesothelioma of the tunica vaginalis. Can. Urol. Assoc. J..

[5] Tan W.K., Tan M.-Y., Gan S.C., Pathmanathan R., Tan H.M. (2016). Well-Differentiated Papillary Mesothelioma of the Tunica Vaginalis: Case Report and Systematic Review of Literature. Clin. Genitourin. Cancer.

[6] Trpkov K., Barr R., Kulaga A., Yilmaz A. (2011). Mesothelioma of tunica vaginalis of “uncertain malignant potential”—An evolving concept: Case report and review of the literature. Diagn. Pathol..

[7] Brimo F., Illei P.B., Epstein J.I. (2010). Mesothelioma of the tunica vaginalis: A series of eight cases with uncertain malignant potential. Mod. Pathol..

[8] Guney N., Basaran M., Karayigit E., Müslümanoglu A., Guney S., Kilicaslan I., Gulbarut S. (2007). Malignant mesothelioma of the tunica vaginalis testis: A case report and review of the literature. Med. Oncol..

[9] Churg A., Allen T., Borczuk A.C., Cagle P.T., Galateau-Sallé F., Hwang H., Murer B., Murty V.V., Ordonez N., Tazelaar H.D. (2014). Well-differentiated papillary mesothelioma with invasive foci. Am. J. Surg. Pathol..

[10] Shimizu S., Yoon H.-E., Ito N., Tsuji T., Funakoshi Y., Utsumi T., Sakaguchi M., Tsujimura T., Kasai T., Hiroshima K. (2017). A case of solitary well-differentiated papillary mesothelioma with invasive foci in the pleura. Pathol. Int..

[11] Galateau-Sallé F., Vignaud J.M., Burke L., Gibbs A., Brambilla E., Attanoos R., Goldberg M., Launoy G. (2004). Well-differentiated papillary mesothelioma of the pleura: A series of 24 cases. Am J. Surg. Pathol..

[12] Tufano A., Flammia R.S., Antonelli L., Minelli R., Franco G., Leonardno C., Cantisani V. (2021). The value of contrast-enhanced ultrasound (CEUS) in differentiated testicular masses: A systematic review and meta-analysis. Appl. Sci..

[13] Jiang Q., Li F., Yu J., Jiang X.-H., Du L.-F., Bai M., Li Z.-J., Shi Q.-S. (2022). Contrast-enhanced ultrasound a valuable imaging modality for characterizing testicular lesions. Asian J. Androl..

